# Cost Effectiveness of the Use of Prophylactic Mesh To Prevent Parastomal Hernia After Urinary Diversion with an Ileal Conduit

**DOI:** 10.1016/j.euros.2022.03.011

**Published:** 2022-04-21

**Authors:** Sanjib Saha, Ulf Gerdtham, Mats Bläckberg, Petter Kollberg, Fredrik Liedberg

**Affiliations:** a Health Economics Unit, Department of Clinical Sciences (Malmö), Lund University, Lund, Sweden; b Department of Economics, Lund University, Lund, Sweden; c Department of Urology, Helsingborg County Hospital, Helsingborg, Sweden; d Institute of Translational Medicine, Lund University, Malmö, Sweden; e Department of Urology, Skåne University Hospital, Malmö, Sweden

**Keywords:** Cost-effectiveness analysis, Economic evaluation, Bladder cancer, Cystectomy, Ileal conduit, Parastomal hernia, Mesh

## Abstract

**Background:**

Prophylactic lightweight mesh in the sublay position reduced the cumulative incidence of parastomal hernia (PSH) after cystectomy with ileal conduit diversion in a randomised controlled trial.

**Objective:**

To investigate whether the use of prophylactic mesh is cost-effective in comparison to no mesh from the health care provider perspective.

**Design, setting, and participants:**

Data on health care resource utilisation (outpatient care and inpatient care) were obtained for 159 patients included in a randomised trial. The patients underwent surgery at Skåne University Hospital or Helsingborg County Hospital (80 with a prophylactic mesh and 79 without) and information about care was ascertained from the regional health care register. The patients underwent surgery between 2012 and 2017 and were followed until death or August 2020.

**Outcome measurements and statistical analyses:**

The primary outcome measure was the clinical incidence of PSH. Costs are reported in Euro in 2020 prices (€1 = 10.486 Swedish Krona) and presented as the incremental cost-effectiveness ratios (ICERs) with confidence intervals (CIs) calculated using a nonparametric bootstrap procedure. Sensitivity analyses and subgroup analyses were performed to capture the uncertainty for ICERs.

**Results and limitations:**

The mean difference in total costs between the mesh and no-mesh groups was −€2047 (95% CI −€16 441 to €12 348). Seventeen patients (21.5%) in the no-mesh group developed clinical PSH versus six patients (7.5%) in the mesh group (*p* = 0.001). This indicates that mesh is less costly and more effective compared to no mesh from the health care provider perspective. Subgroup analyses showed that results were more advantageous for women and for patients younger than 71 yr and with less comorbidity than for their counterparts.

**Conclusions:**

The use of prophylactic mesh during ileal conduit reconstruction to prevent PSH is cost-effective from the health care provider perspective.

**Patient summary:**

In patients having their bladder surgically removed, a mesh implant can be inserted when a portion of the intestine is used to create an opening to drain urine from the body. Our results show that mesh use to prevent development of a hernia at the opening where urine exits the body is cost-effective from the perspective of health care providers.

## Take Home Message

The use of prophylactic mesh is cost-effective in reducing the risk of parastomal hernia after ileal conduit diversion.

## 1 Introduction

Parastomal hernia (PSH) is a common complication after stoma creation that leads to a decrease in quality of life for patients and increases in health care costs [1]. Meta-analyses have shown that use of a prophylactic mesh reduces the rate of PSH [2], [3] for patients receiving end colostomies. In addition, a prospective randomised multicentre study showed that prophylactic implantation of a lightweight mesh decreased the risk of PSH when constructing an ileal conduit in comparison to no mesh [4]. However, decision-makers need to know whether prophylactic mesh in this setting represents good use of limited resources and is cost-effective before its adoption in routine clinical practice for patients receiving ileal conduit diversion. Cost-effectiveness analyses (CEAs) are central to national reimbursement decisions on new health technologies or treatment methods both in general and for high-income countries such as Sweden [5]. Informed and transparent decision-making is important for efficient allocation of scarce health care resources. A previous study on the use of mesh prophylaxis to prevent PSH showed that this is cost-effective for patients undergoing permanent colostomy for rectal cancer [6]. In another CEA, synthetic mesh was the most cost-effective approach in preventing PSH when compared to a biological mesh and no mesh for patients undergoing end-colostomy creation during rectal cancer surgery [7]. However, to the best of our knowledge there has been no research on the cost-effectiveness of mesh in preventing PSH in patients receiving an ileal conduit. Thus, the objective of this study was to perform a CEA on the use of prophylactic mesh to prevent PSH after ileal conduit diversion from a health care provider perspective in Sweden.

## 2 Patients and methods

### 2.1 Patients

Between 2012 and 2017, 242 patients undergoing open radical cystectomy and ileal conduit diversion were randomised 1:1 to prophylactic mesh insertion in the intervention arm and conventional conduit construction in the control arm in three different hospitals in Sweden (ISRCTN 95093825). The inclusion criteria were cystectomy and ileal conduit in patients aged ≥18 yr and the absence of a previous stoma. The exclusion criteria were a previous stoma and lack of informed consent.

In the experimental arm a lightweight mesh was placed in a sublay position between the rectus abdominis muscle and the posterior rectus sheath, and the conduit was brought out through a cross-shaped incision in the centre of the mesh. The mesh was anchored to the posterior rectus sheath with 2-0 polydioxanone (PDS) sutures in each corner. The conduit was fixed at the 6- and 12-o’clock positions to the anterior rectus sheath with a 4-0 resorbable suture in both the control arm and the intervention arm, and three monofilament 4-0 resorbable sutures were also used to evert and mature the ileal conduit in all patients. Further details of the surgery performed and of the trial, including the clinical effectiveness of the intervention, can be found elsewhere [4].

**Fig. 1:**
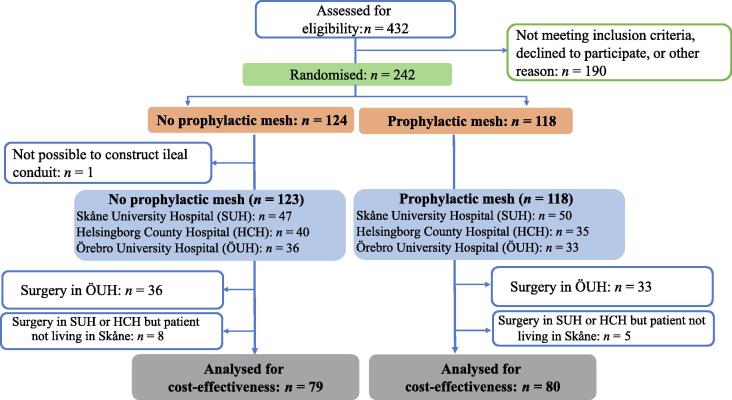
Consolidated Standards of Reporting Trials (CONSORT) diagram describing the study population.

As the cost data were from the Skåne County Council register, patients undergoing surgery in the one hospital (Örebro University Hospital) not maintained by Skåne County Council were excluded from the analysis. Thus, of the 242 patients originally randomised in the trial, only the 159 (mesh *n* = 80, no mesh *n* = 79) whose surgery was performed in Skåne University Hospital or Helsingborg County Hospital were included in the CEA. A further 13 patients were excluded as they were referred from hospitals outside Skåne County and information on their health care consumption was missing from the Skåne County Council database (Fig. 1).

The trial was approved by the Research Ethics Board of Lund University (2012/336).

### 2.2 Cost measures

The current CEA was performed from a health care provider perspective, including costs for both inpatient and outpatient care at the hospitals in Skåne county as the relevant geographic area where the trial patients reside. Data on health care utilisation were obtained from the Skåne health care registers, from which costs were computed per patient on the basis of diagnosis-related group (DRG) codes [8]. DRG is a patient classification system used to reimburse health care providers on the basis of the expected resource intensity of care. DRGs are assigned according to principal diagnosis, secondary diagnosis, surgical procedure performed, comorbidities and complications, and patient’s age, sex, and discharge status [9]. The costs are based on a cost-per-patient system that contains the actual individual cost data for each participant in the trial. The costs for mesh and the additional two sutures (2-0 PDS using a CT-2 needle) used to anchor the mesh were obtained from the actual hospital costs after procurement (internal article identification numbers 102122 and 37016, respectively). We gathered cost data from the date of cystectomy up to August 2020. Costs for primary care are not included since the local county council does not record the cost-per-patient for primary care. Costs are measured in Swedish kronor (SEK) at 2020 prices and converted to Euro (€1 = 10.486 SEK) [10].

### 2.3 Effect measures

We used the primary outcome of the trial, which was the incidence of clinical PSH during follow-up [10]. Clinical PSH was assessed at follow-up visits at 6, 12, and 24 mo postoperatively, as well as at later visits scheduled at the discretion of the treating urologist. Clinical PSH incidence was registered without any a priori definitions applied for clinical PSH, and both symptomatic and asymptomatic findings were reported. Since this is a dichotomous variable (yes/no), we present the outcomes as percentages and estimated the probability of having PSH to achieve individual variations. To this end, we performed multivariable logistic regression for which PSH incidence was the dependent variable (yes/no) and the independent variables were age, sex, and prophylactic mesh status (mesh/no mesh).

### 2.4 Statistical analyses

Statistical analyses were performed to assess differences between the mesh and no-mesh groups using t tests for continuous variables, χ^2^ tests for dichotomous variables, and Fisher’s exact test for PSH incidence. We also performed linear and logistic regressions in sensitivity analyses controlled for the variables that might influence costs and PSH for our investigation of a subsample of the randomised trial. Analyses were conducted using Stata/SE 15.1 (StataCorp LP, College Station, TX, USA) [11].

### 2.5 Analysis of cost-effectiveness

Cost-effectiveness was estimated as the incremental cost-effectiveness ratio (ICER), which is the ratio of the difference in average costs per patient to the difference in health effects per patient $\left(ICER=\;\frac{{Cost}_{mesh}-\;{Cost}_{no\;mesh}}{{Effects}_{mesh}-{Effects}_{no\;mesh}}\right)$ for the mesh group compared to the no-mesh group. Sampling uncertainty was assessed using 5000 bootstrap resamples to estimate the ICERs [12]. These bootstrapped ICERs and point estimates are graphically presented on a four-quadrant cost-effectiveness plane (CE-plane), with effect differences plotted on the *x*-axis and cost differences on the *y*-axis between the mesh and no-mesh groups [13]. We present the effect differences as the probability of not developing PSH. Both the southeast (SE) and northwest (NW) quadrants of the CE-plane explicitly indicate whether an intervention is cost-effective (dominant if the ICER is located in the SE) or not (dominated if the ICER is located in the NW) in relation to its comparator. However, cost-effectiveness is difficult to determine if the ICER is located either in the southwest (SW) or northeast (NE) quadrant. ICER in the NE quadrant reflects a more effective and more costly intervention than the comparator, and whether an intervention in the NE quadrant is cost-effective depends on societal willingness to pay (WTP), which is how much society is willing to pay to receive an additional health effect [14]. The CEA was conducted according to the Consolidated Health Economic Evaluation Reporting Standards (CHEERS) guideline [15].

### 2.6 Base case analysis

The base-case CEA includes all the costs (inpatient, outpatient, operation time, and intervention costs) and clinical PSH incidence as the outcome.

### 2.7 Sensitivity analyses and subgroup analyses

Several sensitivity and subgroup analyses were performed to capture uncertainties around the base-case estimate.

#### 2.7.1 Controlling for variables that might affect costs

We performed multivariable linear regression analysis to control for variables that might affect costs. These variables were smoking status, American Society of Anesthesiology (ASA) score, gender, operating hospital, body mass index (BMI), use of preoperative chemotherapy, follow-up duration, previous midline laparotomy, and treatment (mesh vs no-mesh group) in the first scenario (scenario 1a), and then controlled for the significant variables, which were ASA score and follow-up duration in scenario 1b.

#### 2.7.2 Controlling for variables that might affect the risk of PSH

We used the same variables as in scenario 1a to control for the effect on risk of PSH. However, we performed multivariable logistic regression analysis with PSH incidence (yes/no) as the dependent variable.

#### 2.7.3 Removal of cost outliers

To exclude low-cost and high-cost outliers for health care costs, participants with the top 5% costs and bottom 5% costs were excluded from the analysis.

#### 2.7.4 Subgroup analysis by sex

Owing to the higher risk of PSH among women [16], we stratified the results by sex.

#### 2.7.5 Subgroup analysis by ASA score

The results were also stratified by ASA score since the costs and health effects may be affected by comorbidity. We merged patients with ASA scores of III and IV together into one class.

#### 2.7.6 Subgroup analysis by overweight status

The results were stratified by overweight status on the basis of hypothesis-generating findings suggesting a higher risk of PSH for individuals with high BMI [16]. We used BMI of 25 kg/m^2^ as the threshold for overweight according to the World Health Organization [17].

#### 2.7.7 Subgroup analysis by age

The results were also stratified by age (≤71 yr vs ≥72 yr), with the cutoff for dichotomisation chosen according to the median age at cystectomy in Skåne county during 2016–2020, which was 71 yr [18].

## 3 Results

### 3.1 Patients and surgical characteristics

Even though this CEA is based on the subgroup of patients residing in Skåne county who participated in the original trial, there were no significant differences in patient characteristics between the two groups except for operation time (Table 1). On average, operation time was longer for the mesh group (*p* = 0.003) than for the no-mesh group.

**Table 1:** Characteristics of patients participating in the trial stratified by mesh receipt

Parameter	Mesh	No mesh	*p* value
	(*n* = 80)	(*n* = 79)	
Median age, yr (interquartile range)	72 (66–77)	74 (68–79)	0.34
Gender, *n* (%)			0.96
Male	62 (78)	61 (77)	
Female	18 (22)	18 (23)	
American Society of Anesthesiologists score, *n* (%)			0.97
I	10 (13)	10 (13)	
II	44 (55)	44 (56)	
III–IV	26 (32)	24 (31)	
Smoking status, *n* (%)			0.45
Nonsmoker	20 (27)	15 (21)	
Previous smoker	36 (49)	42 (59)	
Current smoker	18 (24)	14 (20)	
Median body mass index, kg/m^2^ (interquartile range)	26 (22–28)	26 (23–28)	0.98
Median follow-up, mo (IQR)	30 (2–58)	21 (6–50)	0.19
Median time to clinical PSH, mo (interquartile range)	9 (6–14)	14 (7–27)	0.23 a
Operating hospital, *n* (%)			0.48
Skåne University Hospital	45 (56)	40 (51)	
Helsingborg County Hospital	35 (44)	39 (49)	
Median operation time, min (interquartile range)	420 (364–500)	411 (340–480)	0.033
Survival status, *n* (%)			0.37
Dead	24 (30)	29 (37)	
Alive	56 (70)	50 (63)	
Neoadjuvant or induction chemotherapy, *n* (%)			0.45
Yes	47 (59)	51 (65)	
No	33 (41)	28 (35)	
90-d Clavien complications, *n* (%)			0.73
Grade <3	12 (37)	10 (33)	
Grade ≥3	20 (63)	20 (67)	
Adjuvant chemotherapy, *n* (%)			0.55
Yes	5 (6)	7 (9)	
No	74 (94)	72 (91)	

PSH = parastomal hernia.

a Mann-Whitney test.

### 3.2 Costs and effects

The mean inpatient costs were €60 726 and €63 811 and the mean outpatient costs were €22 337 and €23 758 for the mesh and no-mesh groups, respectively (Table 2). There were no significant differences in total, inpatient, or outpatient costs, but there was a significant difference in costs related to operation time. The operation time cost was €598 higher for the mesh group than for the no-mesh group (*p* = 0.032). The mean total cost per patient was €69 837 in the mesh group and €71 884 in the no-mesh group (Table 2), with a nonsignificant cost difference of −€2047 (95% CI −€16 441 to €12 348). Cumulative PSH incidence was lower in the mesh group (*n* = 7/80, 8%) than in the no-mesh group (*n* = 17/79, 22%; *p* = 0.014; Table 3). The number needed to treat to prevent one PSH case was seven patients. No long-term complications related to mesh use in the intervention group, such as mesh infections or erosions, were observed during follow-up. One patient in the mesh group and three patients in the no-mesh group required surgical PSH repair.

**Table 2:** Differences in pooled mean cost

Cost item	Pooled mean cost ± standard error (€)		Difference, € (95% CI)
	Mesh (*n* = 80)	No mesh (*n* = 79)	
Inpatient costs	38 389 ± 3452	40 053 ± 4611	−1664 (−12 988 to 9659)
Outpatient costs	22 337 ± 1946	23 758 ± 3150	−1421 (−8648 to 5806)
Inpatient and outpatient costs	60 726 ± 4333	63 811 ± 5909	−3085 (−17 478 to 11 307)
Operation	8448 ± 202	7847 ± 190	598 (53–1144)*
Mesh and two extra sutures	613		
Total cost	69 837 ± 4350	71 884 ± 5893	−2047 (−16 441 to 12 348)

CI = bootstrapped confidence interval.

* *p* = 0.032 (independent-sample t test).

**Table 3:** Differences in the cumulative incidence of clinical parastomal hernia

	Mesh (*n* = 80)	No mesh (*n* = 79)	Difference
Parastomal hernia (*n*)			
Yes	6	17	−11 a
No	74	62	
Percentage	7.50	21.52	−14.02
Predicted probability b	0.073	0.214	−0.140 c

a *p* = 0.014 (Fisher’s exact test).

b Predicted probability was estimated using multivariable logistic regression with age, sex, and mesh/no mesh as independent variables.

c *p* = 0.001 (t test).

### 3.3 Base-case, sensitivity, and subgroup analyses

**Fig. 2:**
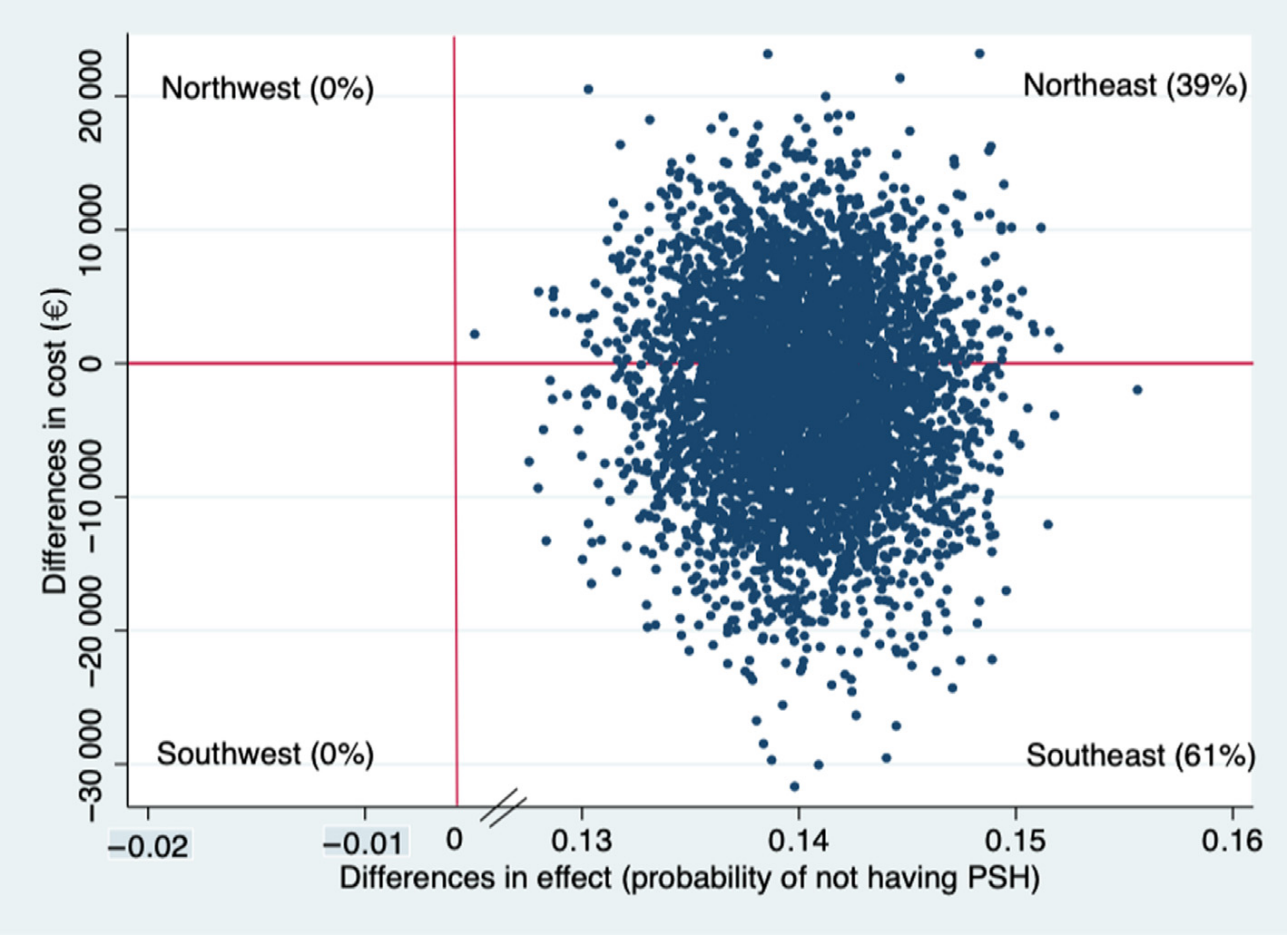
Cost-effectiveness plane from the health care provider perspective.

For the base-case analysis, the mesh group dominated the no-mesh group: the intervention was less costly and more effective. On the CE-plane, 61% of the pairs were in the SE quadrant (less costly and more effective), followed by 39% in the NE quadrant (more costly and more effective; Fig. 2). The base-case results were not sensitive to any of the scenarios except when the cost outliers were removed (Table 4). The ICER was no longer dominant and was €12 per percentage point change in cumulative PSH incidence. The base-case result was sensitive to several subgroup analyses (Table 4). For men, the ICER was not dominant and was €180 per percentage point change in cumulative PSH incidence; in other words, the cost to prevent one case on incident PSH is €18 000. Similarly, the ICERs were not dominant for patients with an ASA score >I. For elderly patients (≥72 yr) the ICER was €31 500 per percentage point for the cumulative PSH incidence.

**Table 4:** Mean differences in cost and outcomes and the resulting ICER

No.	Scenario	Cost difference (€)	Effect difference (%)	ICER
Base-case analysis				
	All health care costs	−2047	−14.02	Dominant
Sensitivity analyses				
1a	Cost-adjusted^a^	−5745	−14.02	Dominant
1b	Cost-adjusted^b^	−4892	−14.02	Dominant
2	Effect-adjusted^a^	−2047	−15.32	Dominant
3	Removal of 5% cost outliers (74/71)^c^	175	−14.43	12
Subgroup analyses				
4	Sex			
	Female (18/18)^c^	−18 211	−11.11	Dominant
	Male (62/61)^c^	2673	−14.88	180
5	ASA class			
	ASA I (10/10)^c^	−35 603	−30.00	Dominant
	ASA II (44/44)^c^	1148	−13.63	84
	ASA III–IV (26/24)^c^	4595	−8.79	523
6	Body mass index			
	Not overweight (34/37)^c^	−2750	−14.03	Dominant
	Overweight (46/42)^c^	−2880	−15.12	Dominant
7	Age			
	≤71 yr (27/31)^c^	−9256	−25.19	Dominant
	≥72 yr (53/48)^c^	2286	−7.26	315

ASA = American Society of Anesthesiologists; ICER = incremental cost-effectiveness ratio.

a Adjusted for smoking, ASA class, gender, operating hospital, body mass index, use of preoperative chemotherapy, follow-up duration, and previous midline laparotomy.

b Adjusted for ASA class and follow-up duration.

c Sample size for mesh/no mesh in parentheses.

## 4 Discussion

We have performed cost-effectiveness analyses (CEA) of using prophylactic mesh to prevent PSH after ileal conduit diversion from a health care provider perspective. The use of a prophylactic mesh reduced cost (although not statistically significant) and the incidence of PSH. The cost-effectiveness plane also indicates that 61% of the cost-effect pairs were in the southeast quadrant. The subgroup analyses revealed that in men to prevent one PSH, health care needs to spend €18 000. Since there is no established threshold on how much society is willing to pay to prevent one PSH, it is difficult to interpret this finding as cost-effective. Similarly, in the subgroups with ASA score above II and patients aged 72 yr of age and above the ICERs were not dominant. Consequently, these findings indicate that using mesh in women, in patients with ASA class I and in patients younger than 71 yr are more beneficial than their corresponding counterparts, respectively.

Since our study uses a patient cohort from the first randomised trial examining the use of prophylactic mesh to prevent PSH in patients receiving an ileal conduit and hence is the first CEA in this setting, it is difficult to compare the findings with similar studies. However, researchers in Canada reported that a prophylactic mesh was dominant in preventing PSH in patients with rectal cancer receiving a permanent colostomy, which is in line with our findings [6]. They also found that mesh was not dominant in advanced disease (rectal cancer stage IV), a result also in line with our observation for patients with more advanced comorbidity (ASA score ≥II).

The main study limitation is the lack of statistical power, as the power calculation was conducted from the clinical outcome perspective instead of a CEA perspective. A post hoc power calculation for cost-effectiveness using cost and outcome data from the present trial was therefore conducted [19], [20]. As mentioned earlier, cost-effectiveness depends on societal WTP, and there is no agreed WTP threshold for preventing incidence of one PSH case. For WTP thresholds ranging from €100 to €100 000, the power calculation showed that, a sample size ranging from eight to 244 patients per group would be needed. Therefore, a CEA based on a simulation model in which data from this and/or similar future randomised trials could be included might be a more suitable approach for evaluating the cost-effectiveness.

Another limitation of the present study is that the trial did not include any PSH-specific outcome measures, such as hernia-related problems with stoma appliances or local pain; however, only a minority of patients with PSH are asymptomatic [21]. Furthermore, the lack of a more structured definition for clinical PSH, such as the European Hernia Society stratification of PSH based on hernia size that was applied in the aforementioned study [21], is a study limitation. Similarly, no generic or disease-specific instrument for measuring patient quality of life was available for the CEA. A generic instrument such as the European Quality of Life 5 Dimension (EQ-5D) would have been helpful in estimating quality-adjusted life year (QALY) [22] gains for patients receiving a mesh. QALYs not only capture patient quality of life resulting from the intervention of interest but also facilitate its comparison with other similar or different interventions in terms of cost-effectiveness. Furthermore, the lack of cost estimates from primary care and medication costs are study limitations. However, since the patients underwent surgery in a regionalised cystectomy setting in two hospitals, it is reasonable to assume that they would contact the specialised unit rather than their primary health care centre in the case of any complications or health-related issues. To the best of our knowledge, this study is the first CEA conducted in this setting, and the preciseness of the cost data and the randomised controlled study design add strength to the results.

## 5 Conclusions

The use of prophylactic mesh is cost-effective in reducing the risk of PSH after ileal conduit diversion when compared to standard care. A CEA based on larger randomised trials or a simulation model incorporating patient quality of life would further validate these findings.

  ***Author contributions***: Fredrik Liedberg had full access to all the data in the study and takes responsibility for the integrity of the data and the accuracy of the data analysis.

*Study concept and design*: Saha, Gerdtham, Bläckberg, Liedberg.

*Acquisition of data*: Saha, Gerdtham, Bläckberg, Liedberg.

*Analysis and interpretation of data*: Saha, Gerdtham, Liedberg, Bläckberg, Kollberg.

*Drafting of the manuscript*: Saha, Gerdtham, Liedberg, Bläckberg, Kollberg.

*Critical revision of the manuscript for important intellectual content*: Saha, Gerdtham, Liedberg, Bläckberg, Kollberg.

*Statistical analysis*: Saha.

*Obtaining funding*: Liedberg, Bläckberg, Kollberg.

*Administrative, technical, or material support*: Liedberg, Kollberg.

*Supervision*: Liedberg, Gerdtham.

*Other*: None.

  ***Financial disclosures:*** Fredrik Liedberg certifies that all conflicts of interest, including specific financial interests and relationships and affiliations relevant to the subject matter or materials discussed in the manuscript (eg, employment/affiliation, grants or funding, consultancies, honoraria, stock ownership or options, expert testimony, royalties, or patents filed, received, or pending), are the following: None.

  ***Funding/Support and role of the sponsor*:** This work was supported by the Swedish Cancer Society (CAN 2020/0709), Lund Medical Faculty (ALF) including funding to the Health Economic Unit (2018-Project 0160), Skåne University Hospital Research Funds, the Gyllenstierna Krapperup Foundation, the Skåne County Council Research and Development Foundation (REGSKANE-821461), the Cancer Research Fund at Malmö General Hospital, the Gösta Jönsson Research Foundation, the Foundation of Urological Research (Ove and Carin Carlsson Bladder Cancer donation), the Bergqvist Foundation, the Stig and Ragna Gohrton Research Foundation, the Thelma Zoega Research Foundation, and the Hillevi Fries Research Foundation. The sponsors had no direct role in the study.
